# Anti-Vascular Endothelial Growth Factor Agents for Ocular Angiogenesis and Vascular Permeability

**DOI:** 10.1155/2012/898207

**Published:** 2012-03-18

**Authors:** Toshiaki Kubota, Yoshiaki Kiuchi, Carl Sheridan

**Affiliations:** ^1^Department of Ophthalmology, Faculty of Medicine, Oita University, Oita, Japan; ^2^Department of Ophthalmology and Visual Science, Graduate School of Biomedical Sciences, Hiroshima University, Hiroshima, Japan; ^3^Department of Eye and Vision Science, Institute of Ageing and Chronic Disease, University Clinical Departments, University of Liverpool, Liverpool, UK

Recent clinical trials regarding the intravitreal injection of antivascular endothelial growth factor (VEGF) agents (ranibizumab, bevacizumab, pegaptanib, and aflibercept) have shown excellent results in the treatment of angiogenic pathologies including choroidal neovascularization, macular edema, proliferative diabetic retinopathy, and neovascular glaucoma. Ranibizumab (Lucentis, Genectech, San Francisco), a fragment of a humanized monoclonal antibody against all VEGF isoforms, is beneficial in the treatment of choroidal neovascularization secondary to age-related macular degeneration (AMD). Bevacizumab (Avastin, Genentech, San Francisco), a humanized, recombinant monoclonal IgG antibody that binds and inhibits all VEGF isoforms, has been approved as an adjuvant agent for the treatment of colorectal carcinoma and has also been increasingly used as an offlabel therapy in the field of ophthalmology. Pegaptanib (Mucugen, Pfizer, New York) is a 28-base ribonucleic acid aptamer, covalently linked to two branched 20-kD polyethylene glycol moieties, that was developed to bind and block the activity of extracellular VEGF, specifically the 165-aminoacid isoform (VEGF165). Aflibercept (VEGF Trap-Eye, Regeneron, New York; Bayer, Berlin, Germany) is a 115-kDa recombinant fusion protein consisting of the VEGF-binding domains of human VEGF receptors 1 and 2 fused to the Fc domain of human immunoglobulin-G1.

 Intraocular injections of anti-VEGF drug are considered to be an effective treatment for macular edema after retinal vein occlusion. The recent reports may lead to a shift in treatment paradigm for diabetic macular edema, from laser photocoagulation to newer approaches using anti-VEGF drugs. There have been several well-publicized prospective, randomized studies that demonstrated the efficacy of intravitreal injection of anti-VEGF drugs for patients with AMD. Adjuvant bevacizumab for neovascular glaucoma may prevent further peripheral anterior synechiae formation, and it is likely to open up a therapeutic window for a panretinal photocoagulation and trabeculectomy. Intravitreal injection of bevacizumab (IVB) results in a substantial decrease in bleeding from the retinal vessels or new vessels during a standard vitrectomy. IVB has also been reported to be effective for inducing the regression of new vessels in proliferative diabetic retinopathy. The use of bevacizumab in stage 4 or 5 retinopath of prematurity (ROP) is to reduce the plus sign to help reduce hemorrhage during the subsequent vitrectomy. Some authors reported cases of resolution of stage 4A ROP after bevacizumab injection.

 This special issue of anti-VEGF agents comprises five review articles and two research articles. The review article of K. Kimoto and T. Kubota is a concise overview on the application of anti-VEGF agents in angiogenic pathologies and vascular permeability. The review article of A. Willard and I. Herman describes the mechanisms behind diabetic retinal complications, current research supporting anti-VEGF medications, and future therapeutic directions. The review of P. Keane and S. Sadda summarizes the history of anti-VEGF drugs and presents a guide for clinicians on the development of anti-VEGF therapies for intraocular use. The review article of Y. Park et al. describes the treatment possibilities including combination therapy for age-related macular degeneration. The review article of I. Zampros et al. summarizes the current literature regarding the effectiveness of current anti-VEGF treatment for neovascular AMD. The two research articles describe (i) bevacizumab injection in patients with age-related macular degeneration associated with poor initial visual acuity, (ii) photodynamic therapy combined with intravitreal ranibizumab for exudative age-related macular degeneration. We sincerely hope that the present volume of a special issue series may provide useful information to understand the mechanisms of the clinical effects of anti-VEGF drug therapy, as well as the evaluation of their outcomes for the readers in Journal of Ophthalmology.

**Toshiaki Kubota** **Toshiaki Kubota** **Yoshiaki Kiuchi** **Yoshiaki Kiuchi** **Carl Sheridan** **Carl Sheridan**

